# A pattern of cerebral perfusion anomalies between Major Depressive Disorder and Hashimoto Thyroiditis

**DOI:** 10.1186/1471-244X-11-148

**Published:** 2011-09-13

**Authors:** Maria Carolina Hardoy, Mariangela Cadeddu, Alessandra Serra, Maria Francesca Moro, Gioia Mura, Gisa Mellino, Krishna M Bhat, Gianmarco Altoé, Paolo Usai, Mario Piga, Mauro G Carta

**Affiliations:** 1 Department of Psychiatry, Reald University, Vlore, Albania; 2 Department of Public Health, University of Cagliari, Cagliari, Italy; 3 Department of Internal Medicine, University of Cagliari, Cagliari, Italy; 4 Department of Neuroscience & Cell Biology, University of Texas Medical Branch, Galveston, Texas, USA; 5 Department of Psychology, University of Cagliari, Cagliari, Italy

## Abstract

**Background:**

This study aims to evaluate relationship between three different clinical conditions: Major Depressive Disorders (MDD), Hashimoto Thyroiditis (HT) and reduction in regional Cerebral Blood Flow (rCBF) in order to explore the possibility that patients with HT and MDD have specific pattern(s) of cerebral perfusion.

**Methods:**

Design: Analysis of data derived from two separate data banks.

Sample: 54 subjects, 32 with HT (29 women, mean age 38.8 ± 13.9); 22 without HT (19 women, mean age 36.5 ± 12.25).

Assessment: Psychiatric diagnosis was carried out by Simplified Composite International Diagnostic Interview (CIDIS) using DSM-IV categories; cerebral perfusion was measured by ^99 m^Tc-ECD SPECT. Statistical analysis was done through logistic regression.

**Results:**

MDD appears to be associated with left frontal hypoperfusion, left temporal hypoperfusion, diffuse hypoperfusion and parietal perfusion asymmetry. A statistically significant association between parietal perfusion asymmetry and MDD was found only in the HT group.

**Conclusion:**

In HT, MDD is characterized by a parietal flow asymmetry. However, the specificity of rCBF in MDD with HT should be confirmed in a control sample with consideration for other health conditions. Moreover, this should be investigated with a longitudinally designed study in order to determine a possible pathogenic cause. Future studies with a much larger sample size should clarify whether a particular perfusion pattern is associated with a specific course or symptom cluster of MDD.

## Background

Hashimoto Thyroiditis [HT] is a chronic organ-specific autoimmune disorder commonly observed in clinical practice and is frequently associated with mood disorders [1-3]. In patients with HT, a form of encephalopathy known as Hashimoto Encephalopathy (HE) has been described as a severe but rare syndrome with different clinical presentations and course [4]. Presentation of such a syndrome may include alteration of conscious level, seizures, tremor, myoclonus, ataxia, or multiple stroke-like episodes. Psychiatric symptoms, including depression and psychosis, have also been reported [5]. A manic episode associated with HT, pathological EEG and response to short-term treatment with high doses of prednisolone, has been reported as the first case of bipolar disorder due to HE [6].

The aetiopathogenesis of HE is not yet well defined. This form of encephalopathy is understood to be independent from thyroid function since patients can present with variable clinical pictures from frank hypothyroidism to hyperthyroidism, but subclinical hypothyroidism is more frequent [7]. However, independent of these rare cases of HE, several observations indicate that there is a frequent decrease in cerebral perfusion in patients with autoimmune thyroiditis. This suggests cerebral vasculitis as a possible pathogenic model or cause [8,9].

A previous study by Zetting et al. [8] indicated a reduced cerebral perfusion with SPECT in patients suffering from HT. This study did not show any specific topographic pattern of hypoperfusion with SPECT-VOI based analysis, however, it did show that the left posterior gyrus of the cingulate region was most affected. Another study by Piga et al. [9] showed a high prevalence of mild, yet significant perfusion anomalies in cerebral cortex of HT patients with euthyroidism, however, this was not observed in patients with non-toxic multinodular goitre. These perfusion anomalies are qualitatively similar to those observed in sporadic cases of severe HE. This survey additionally found a frontal perfusion asymmetry in patients with HT compared to patients with non-toxic multinodular goitre.

The use of functional imaging methods for studying psychiatric disorders have aroused a great deal of interest over the past few years, both for clinical (diagnostic and therapeutic) and research purposes [10-12]. These studies showed that in patients with depression, ventral frontal and prefrontal regions had an increase in metabolism or perfusion, whereas more rostral regions within the cingulate gyrus and dorsolateral prefrontal cortex had a decrease in perfusion/metabolism [13-19].

Recently Bocchetta et al. [20] described a case of affective psychosis with HT and brain perfusion abnormalities. They hypothesized that abnormalities in cortical perfusion might represent a pathogenic link between HT and mood disorders, even in the absence of other prominent symptoms of CNS inflammation or EEG abnormalities. Therefore, the rare severe cases of HE presenting with mood disorders may represent only the tip of an iceberg.

Considering the possibility of an association between depressive disorders and HT and the association between decreases in rCBF and depressive disorders, the hypothesis that the three conditions (decreases in specific regional blood flow, MDD and HT) might be interrelated is of great interest. This is particularly so if cerebrovascular damage can be invoked in the pathogenesis of psychiatric disorders, such as depression, given that these are often associated with thyroiditis.

In this paper, we evaluated data from\data banks of cerebral perfusion measured using SPECT with ^99 m^Tc etil-cysteinate dimer (ECD) in HT patients with and without any mood disorders, celiac disease with and without mood disorders, and non-toxic nodular goitre with and without mood disorders. While the data bank was not specifically built to investigate correlation between cerebral perfusion, MDD and HT, with appropriate statistical tools to correct confounding factors, we sought to identify correlation, if any, at least from a preliminary heuristic perspective.

## Methods

### Study design

The study adopted a cross sectional observational design. We compared cerebral perfusion through a multivariate data analysis technique in a group of patients with a large proportion affected by HT.

The study made use of collection of data from two separate databases; the data represents investigation of cerebral perfusion and psychiatric disorders in patients with thyroiditis (versus endemic goitre) and in subjects with coeliac disease (with and without thyroiditis). For this reason a significant number of subjects among patients (with HT) and controls (without HT) were affected by coeliac disease; this condition is extremely frequent in HT (Odds Ratio from 5 to 15 in various studies) [21], and will be treated as a confounding variable as per the multivariate data analysis technique.

Patients and control subjects with Coeliac Disease undertook a gluten free diet for at least six months. The duration of symptoms of their coeliac disease varied from 6 months to 52 years, with a mean duration of 14 years (mean ± SD = 14.0 ± 8.3).

Control subjects not affected by coeliac disease had endemic goitre (NTMG), a thyroid condition not dependent on autommunity and were in euthymic state.

### Sample

The study population included 4 subgroups, all recruited from among inpatients at the University Hospital "Policlinico di Monserrato" of Cagliari; stratification by age implied a subdivision of patients of each subgroup into four age groups: 18-24, 25-44, 45-64 and > 65 years (Table 1).

**Table 1:** The study sample

	Subgroup A HT	Subgroup B **HT and Coel**.	Subgroup D NTM (No HT)	Subgroup C **Coel**. (No HT)
**N**	16	16	6	16

**N. Female**	16	13	6	13

**Mean Age ± sd**	36.6 ± 10.9	43.1 ± 16.1	38 ± 12.5	35.9 ± 12.5

**Age 18 - 24**	1	1	1	1

**Age 25 - 44**	11	7	2	12

**Age 45 - 64**	4	6	3	3

**Age > 65**	0	2	0	0

HT = Hashimoto Thyroiditis; Coel. = Coeliac Disease; TMG: Non-Toxic Multinodular Goitre.

• Subgroup A cases: 16 adults, all female, affected by HT with euthyroidism, aged from 24 to 62 years (mean ± SD = 36.6 ± 10.9).

• Subgroup B, cases with celiac disease: 16 adults affected by coeliac disease and HT with euthyroidism; 13 were women; age ranged from 23 to 77 years; (43.1 ± 16.1).

• Subgroup C, controls: 6 adults with non-toxic nodular goitre (NTNG). All were female, age ranged from 24 to 51 years (38 ± 12.5).

• Subgroup D, controls with celiac disease: 16 adult coeliac patients without HT. Includes 13 women and 3 men, age ranged from 20 to 64 years (35.9 ± 12.5); they are age- and gender-matched with coeliac patients with HT (group B).

### Endocrinological Diagnosis:*Thyroid function tests and ultrasound*

The diagnosis of HT with euthyroidism was made on the basis of coexistence of high titres of antithyroid autoantibody (AbTPO), a marked and diffuse ultrasonographic hypoechogenicity of the thyroid gland and normal concentration of free thyroid hormones and Thyrotropin Stimulating Hormone (TSH).

The diagnosis of non-toxic multinodular goitre (NTMG) was based on ultrasound evidence of one or more thyroid nodules in a normoechogenic gland, along with the absence of antithyroid autoantibodies and normal levels of free thyroid hormones and TSH.

The serum concentration of TSH was measured by chemiluminescence (Ortho-Clinical Diagnostic, Amersham, U.K.) with normal levels ranging from 0.3 to 3 mU/L. Free triiodothyronine (fT3) and thyroxine (fT4) were measured by means of chromatographic method based on the separation of fT4 on Lisophase columns (Technogenetics, Milan, Italy); normal values: fT4: 6.6-1.6 pg/ml; fT3: 2.8-5.6 pg/ml). Antimicrosomal autoantibodies (anti-M) and antithyroglobulin (anti-Tg) were determined through passive agglutination (SERODIA-AMC e SERODIA-ATG; Fujirebio Inc. Pharmaceutical, Tokyo, Japan). The thyroid ultrasound was performed using a "real-time" echograph (ALOKA Mod SSd 500 with a 7,5 Mhz sound). The volumetric levels evaluated with this test were included for all the patients in the study. Given the wide variability of the data, the mean of volumes was calculated and values differing more than 2 standard deviations (SD) were considered abnormal.

### Diagnosis of Coeliac Disease

The diagnosis of coeliac disease in group B and in the control group C was done using the IgA anti-endomysial antibodies (EMA), IgA anti-gliadin antibodies (AGA-A) and small intestine biopsy. EMA were determined by an indirect immunofluorescence method using monkey oesophagus as substrate and titres of ≥ 1:5 were considered positive. AGA-A was measured with an enzyme-linked immunosorbent assay (ELISA) (Phadia Diagnostic, Uppsala, Sweden) with a 10 IU/ml value as the lower positive limit. Biopsy specimens (minimum four) obtained from the distal part of the duodenum, were classified according to the modified Marsh criteria [22]. Specimens were classified as type 0 (normal small bowel mucosa), type 1 (> 40 intra-epithelial lymphocytes [IELs] per 100 enterocytes but without other stigmata of gluten enteropathy), type 3A (mild villous flattening, increase in crypt height, increase in IEL numbers up to > 40 IEL/100 enterocytes), type 3B (marked villous flattening, increase in crypt height, increase in IEL numbers up to > 40 IEL/100 enterocytes) and type 3C (total villous flattening, increase in crypt height, increase in IEL numbers up to > 40 IEL/100 enterocytes)

### Psychiatric Diagnosis

The lifetime psychiatric diagnosis was formulated by physicians using the simplified Italian version of the structured interview CIDIS [23] according to DSM-IV criteria [24]. This tool is the simplified version of CIDI (Composite International Diagnostic Interview) [25]. The CIDIS was administered to all patients soon after their recruitment to the study. The module for the simplified interview CIDIS consists in a total of 105 questions. The interview was done for 45 minutes to about two hours. The presence, duration and severity of symptoms for the various groups were determined.

Collected data were elaborated by a specific computer program, which formulates all the diagnoses according to the Diagnostic and Statistical Manual by the American Psychiatric Association DSM-IV TR [24]. Diagnoses are formulated on a lifetime basis, but it is also possible to relate the diagnosis to four different periods: one week, one month, six months, and one year, respectively.

#### Lifetime diagnosis considered for this study

For the purpose of this study the following specific diagnoses were considered: Major Depressive Disorder (MDD), Panic Disorder (PD), and Generalized Anxiety Disorder (GAD).

### Cerebral Perfusion-SPECT

In order to obtain a bound fraction superior to 90%, ^99 m^Tc etil-cysteinate dimer (ECD) was prepared according to the instructions included in Neurolite kit packaging (Du Pont Pharma, Italy). After 15 minutes from positioning an infusion catether in a forearm vein, with the patient in supine position, with open eyes, and in the absence of any environmental stimuli, intravenous administration of ^99 m^Tc-ECD at a dose of 740 MBq was done. The patient's head was immobilized to avoid any head movements. The cerebral SPECT was carried out after 30 minutes using a double head gamma camera (Varicam- Elscint-Israel) provided with very high resolution collimators. Rotation radius was 13 cm. An acquisition matrix of 128 × 128 pixels with a zoom of 1.0 was used. Data were acquired through 120 scans during 30 sec, each performed at intervals of 3 degrees for a total of 360°. Total time of acquisition was 30 minutes. Images were reconstructed through filtered retroprojections using a Butterworth filter type and a Chang attenuation correction of 0.1/cm ^-1^. Images were evaluated by two nuclear medicine physicians with expertise in the interpretation of cerebral perfusion SPECT. The evaluation of images was both qualitative and semiquantitative. On visual analysis, anomalies in cerebral areas where the uptake of the radioactive drug was decreased in at least three consecutive images, were considered pathological. The semiquantitative analysis in order to evaluate the asymmetries in cortical perfusion was performed through the definition of 3 predetermined regions of interest (ROIs) for each cerebral hemisphere: frontal (F), temporal (T) and parietal (P). The percentage values of asymmetry (Asymmetry Index = AI) were calculated bilaterally for the ROIs to allow the measurement of both the degree and the direction of perfusional asymmetry [26]. The determination of AI was made according the following formula: *[(R-L)/(R+L) × 0, 5] × 100; whereas R = numeric quantitative data of right ROI and L = numeric quantitative data of left ROI. An *AI ± 5% was considered as normal.

Informed, signed consent was obtained from every patient undergoing SPECT.

### Statistical Analyses

Values for continuous data are expressed as mean ± SD. Statistical analyses were carried out in two subsequent steps following a logistic regression approach. In both steps, MDD was considered as dependent variable and all possible risk factors as independent variables.

First, all possible risk factors were entered simultaneously in a single block. For each predictor, a P - value less than 0.05 was considered statistically significant.

Second, all two-way interactions between the possible risk factors were added. To select significant interactions, a backward elimination procedure with a threshold of P < 0.20 was used.

### Ethical Aspects

The two studies generating the two data banks from which the data were drawn were approved by the ethical committee of the Policlinico Universitario di Cagliari. Each subject in the study was identifiable with a code number. An informed consent for the use of anonymous data for scientific purposes was obtained from each patient. The link between the code number and the name of the patient was not available for the researchers. The present study was approved by the ethical committee of the Reald University.

## Results

In Table 2, the frequency, expressed as percentage, of a specific psychiatric diagnosis (MDD, PD and GAD) and at least one psychiatric diagnosis among all the patients (4 sub groups) are shown. No statistically significant differences were detected in the frequency of a specific psychiatric disorder and patients with at least one psychiatric diagnosis in the 4 sub-groups.

**Table 2:** Association between Psychiatric Diagnoses and Subgroups

	Subgroup A HT	Subgroup B **HT and Coel**.	Subgroup C NTMG	Subgroup D **Coel**.	P
**MDD**	37.5% (6)	18.75% (3)	16.67% (1)	25% (4)	0.71

**GAD**	37.5% (6)	37.5% (6)	33.33% (2)	12.5% (2)	0.34

**PD**	12.5% (2)	18.75% (3)	0% (0)	12.5% (2)	0.94

**At least one** **Psychiatric** **Diagnosis**	62.5% (10)	62.5% (10)	33.33% (2)	56.25% (9)	0.71

Observed frequencies are in parentheses. Given that each test was conducted on tables larger than 2 × 2, and because most cells had small counts, Fisher's exact test was used.

Perfusion anomalies in relation to MDD are shown in Table 3. In particular, SPECT Left Frontal hypoperfusion, SPECT Parietal Perfusion Asymmetry, SPECT diffuse hypoperfusion and SPECT Left Temporal showed a significant effect at P < 0.05.

**Table 3:** Perfusion anomalies associated with MDD, resulting from logistic regression with MDD as dependent variable and all the possible risk factors entered in a unique block

Possible Risk Factors	P	O.R	CL 95%
HT	0.94	1.83	0.1 - > 100

SPECT diffuse hypoperfusion	**0.05**	**3.34**	**3.0 - 3.8**

SPECT Left Frontal hypoperfusion	**0.01**	**9.82**	**1.7 - 55.7**

SPECT Right Frontal hypoperfusion	0.82	0.83	0.16 - 4.2

SPECT Left Parietal hypoperfusion	0.85	0.00	-

SPECT Right Parietal hypoperfusion	0.15	4.58	0.1 - 10.2

SPECT Left Temporal hypoperfusion	**0.05**	**5.23**	**1.0 - 26.9**

SPECT Right Temporal hypoperfusion	0.78	0.71	0.1 - 7.2

SPECT Parietal Perfusion Asymmetry (PAI)	**0.03**	**6.8**	**1.2 - 37.4**

P values statistically significant at the 5% level are reported in bold.

In two-way interactions between all possible risk factors, only the interaction between left temporal hypoperfusion and HT reached a statistical significance (p = 0.16; O.R. = 0.07): the presence of HT decreased the significance of left temporal hypoperfusion on MDD.

Parietal perfusion asymmetry was found in 7 patients (21.9%) with HT and in 1 patient (4.5%) without HT. Despite the high O.R (5.9) the difference in frequency does not reach statistical significance (χ^2 ^= 2.0; P = 0.11) (see Table 4). However, within the sub-sample of cases with HT the association between parietal perfusion asymmetry and depression was statistically significant (O.R = 13.1, χ^2 ^= 6.2, P < 0.01) (see table 5) since such an association was not found in subjects without HT (Table 6).

**Table 4:** Parietal perfusion asymmetry in subjects with and without Hashimoto Thyroiditis

	**PAI +**	**PAI -**	
	
**HASHIMOTO THYROIDITIS +**	21.9% (7)	78.1% (25)	**χ**^**2 **^**= 2.0; df = 1** **Yates' correction was used** **P = 0.11** **O.R = 5.9** **CL 95% = 0.7 - 70.8**
	
**HASHIMOTO THYROIDITIS -**	4.5% (1)	95.5% (21)	

**Table 5:** Association between parietal perfusion asymmetry and depression in patients with Hashimoto Thyroiditis (HT+)

	**HT+ PAI+**	**HT+ PAI-**	
	
**DEPRESSED**	71.43% (5)	16% (4)	**χ**^**2 **^**= 6.2; df = 1** **P < 0.01** **Yates' correction was used** **O.R = 13.1** **CL 95% = 1.7 - 98.7**
	
**NOT-DEPRESSED**	28.57% (2)	84% (21)	

**Table 6:** Association between parietal perfusion asymmetry and depression in patients without Hashimoto Thyroiditis (HT-)

	**HT- PAI+**	**HT- PAI-**	
	
**DEPRESSED**	0% (0)	23.80% (5)	**χ**^**2 **^**= 0.44; df = 1** **Yates' correction was used** **P = 0.50** **O.R = 0**
	
**NOT-DEPRESSED**	100% (1)	76.20% (16)	

## Discussion

Our study indicates that the association between MDD and HT (OR = 1.8) is not statistically significant. However, we point out that our sample size is small, and that the control patients had chronic conditions such as coeliac disease and endemic goitre, which have been reported to increase the risk for MDD [27]. One of the aims of the study was to identify specific pattern in MDD associated with HT. However, the fact that the frequency of MDD in the subgroup with HT is not very different from the frequency of MDD in the sub group without HT is only a partial answer given the sample size.

On the other hand, our study indicates that MDD is associated with asymmetry in cerebral diffuse hypoperfusion, right and left Frontal hypoperfusion, right Temporal hypoperfusion and Parietal perfusion. Perfusion anomalies observed in MDD are consistent with results from previous studies [16]. However, left parietal asymmetry is a new finding since it has never been described to our knowledge in association with major depression. Interestingly, this condition of parietal perfusion asymmetry has been described in a study as a perfusion anomalies in post-operative hypothyroidism [28]. Moreover, differential analysis of samples of patients with and without HT, the parietal perfusion asymmetry seems to be associated with MDD in a statistically significant way only in HT compared to control.

Another interesting finding of our study is that left temporal hypoperfusion was associated with MDD, which is consistent with previous studies [16]. On the other hand, the presence of HT decreased the significance of left temporal hypoperfusion on MDD. Consistent with this finding, depression patients with HT did not have any left temporal hypoperfusion.

Ultimately, MDD in HT patients appears to be characterized by an absence of left temporal hypoperfusion and by the presence of parietal perfusion asymmetry, the latter is also typical of non-immune related hypothyroidisms. The thyroiditis cases we studied here were all in fact in euthyroidism condition. This suggests that in the autoimmune thyroditis, a subclinical or slight hypothyroiditis may have a role in MDD even when it is not detectable with routine tests [27]. This can be attributed to the vulnerability of neuronal cells. This finding, if confirmed, would suggest that, although the damage to cerebral vascularisation is associated with a risk for depression, it could be related to thyroid hypofunction as well.

The debate on the pathogenesis of depression in thyroid autoimmunity involves two hypotheses, which might not be mutually exclusive. In one hypothesis, it is suggested that neuronal tissue is hypersensitive to hormonal deficiencies and are more vulnerable to possible subclinical hormonal deficiencies not detectable with routine laboratory tests [27]. In the second hypothesis, a possible pathogenic factor linked to inflammation is postulated, consequent to cytokine activation or extraglandular lesions similar to vasculitis-induced effects [29,30].

The presence of a specific pattern of vascular damage in MDD and HT, with some similarity to the vascular damage observed in autoimmune hypothyrodism, may suggest a causative role for such a damage in the genesis of MDD. Further studies should clarify whether perfusion peculiarities are associated with particular syndromic psychopathological features in MDD concomitant with HT.

It has been recently underlined that thyroid autoimmunity appears to be more frequent in atypical depressions [29] and that these forms would present with differentiated perfusion pictures compared to melancholic depressions. Another notable point is that significantly higher levels of thyroid microsomal antibodies were found in patients with MDD and a family history of dementia, compared with those who did not have such family history [31]. This suggests a specific clinical profile for the association between MDD and autoimmune thyroiditis. The relationship of these clinical peculiarities and specific pattern of vascular damage in MDD and HT need to be further ascertained.

### Study limitations

Our results from this study require further confirmation since the sample we studied, while balanced through multivariate analysis, was not initially selected to evaluate our hypothesis and therefore does not have a control group of MDD without any general medical conditions. However, as previously described, coeliac disease presents a very high risk for HT compared to individuals without thyroid pathologies; thus having eliminated the possible confounding effect due to this factor is not an unremarkable element. Nonetheless, it is necessary to underline that this study may be considered somewhat preliminary.

Another relevant point is that the small sample does not allow us to consider other factors of possible relevance such as 1) the ageing process. It is well known that ageing causes loss of gray matter, and therefore a secondary CBF and metabolism reduction in those same areas can occur, 2) a possible difference in the perfusion in active depressive picture and remitted depressive picture. The rCBF alterations in depression can partially normalize as a response to treatment with medications, interpersonal psychotherapy, or a placebo effect [14,17], but a different pattern of perfusion changes is seen as a response to venlafaxine [18] or cognitive behavioral therapy [19]. Thus these variables need to be considered in further studies. Nevertheless, considering the difficulty and the human, ethical and monetary costs in carrying out studies with SPECT in patients not needing such a test for clinical diagnosis, our preliminary study should be considered important and a basis for future "ad hoc" research.

## Conclusions

This study suggests that in HT, but not in subjects with coeliac disease and euthyroid goitre, the MDD is characterized by temporal hypoperfusion and also by a frequent parietal hypoperfusion asymmetry. Further studies should confirm these results and should clarify whether perfusion peculiarities are associated with the specific syndromic psychopathological features of thyroiditis mood disorders described in the literature.

## Competing interests

The authors declare that they have no competing interests.

## Authors' contributions

MCH participated in the design of the study, in the analysis of the data and drafted the manuscript. MC, AS, MFM, GMu and GMe participated in acquisition of data and critical revision of the manuscript. KMB participated in the design of the study, in the analysis of the data and drafted the manuscript. GA participated in the design of the study and performed the statistical analysis. PU, MP and MGC participated in the design of the study, in the acquisition of data and drafted the manuscript. All authors read and approved the final manuscript.

## Pre-publication history

The pre-publication history for this paper can be accessed here:

http://www.biomedcentral.com/1471-244X/11/148/prepub

## References

[B1] Carta MG Loviselli A Hardoy MC Massa S Cadeddu M Sardu C Carpiniello B Dell'Osso L Mariotti S The link between thyroid autoimmunity (antithyroid peroxidase autoantibodies) with anxiety and mood disorders in the community: a field of interest for public health in the future BMC Psychiatry 2004 4 25 10.1186/1471-244X-4-25 15317653 PMC516779

[B2] Harris B Oretti L Lazarus J Parkes A John R Richards C Newcombe R Hall R Randomised trial of thyroxine to prevent postnatal depression in thyroid-antibody-positive women Br J Psychiatry 2002 180 327 30 10.1192/bjp.180.4.327 11925355

[B3] Fountoulakis KN Kantartzis S Siamouli M Panagiotidis P Kaprinis S Iacovides A Kaprinis G Peripheral thyroid dysfunction in depression World J Biol Psychiatry 2006 7 3 131 7 10.1080/15622970500474739 16861138

[B4] Claussman C Offner C Chevalier Y Sellal F Collard M Encephalopathy and Hashimoto thyroiditis Rev Neurol (Paris) 1994 150 2 166 8 7863160

[B5] Marshall GA Doyle JJ Long-term treatment of Hashimoto's encephalopathy J Neuropsychiatry Clin Neurosci 2006 18 1 14 20 10.1176/appi.neuropsych.18.1.14 16525066

[B6] Müssig K Bartels M Gallwitz B Leube D Häring HU Kircher T Hashimoto's encephalopathy presenting with bipolar affective disorder Bipolar Disord 2005 7 292 7 10.1111/j.1399-5618.2005.00196.x 15898968

[B7] Chong JY Rowland LP Utiger RD Hashimoto encephalopathy: syndrome or myth? Arch Neurol 2003 60 2 164 71 10.1001/archneur.60.2.164 12580699

[B8] Zettinig G Asenbaum S Feuger BJ Hofmann A Diemling M Mittlboeck M Dudczak Increased prevalence of subclinical brain perfusion abnormalities in patients with autoimmune thyroiditis: evidence of Hashimoto's encephalitis? Clin Endocrinol (Oxf) 2003 59 5 637 43 10.1046/j.1365-2265.2003.01901.x 14616889

[B9] Piga M Serra A Deiana L Loi GL Satta L Di Liberto M Mariotti S Brain perfusion abnormalities in patients with euhyroid autoimmune thyroiditis Eur J Nucl Med Mol Imaging 2004 31 12 1639 44 10.1007/s00259-004-1625-7 15290119

[B10] Lewis S Higgins N Brain imaging in Psychiatry 1996 Oxford: Blackwell Science

[B11] Patterson II JC Kotrla KJ Yudofsky S, Hales RE Functional neuroimaging in psychiatry American Psychiatryc Press Textbook of Neuropsychiatry 1997 3 Washington DC: American Psychiatryc Press, Inc.

[B12] Dazzan P Soulsby B Mechelli A Wood SJ Velakoulis D Phillips LJ Yung AR Chitnis X Lin A Murray RM McGorry PD McGuire PK Pantelis C Volumetric Abnormalities Predating the Onset of Schizophrenia and Affective Psychoses: An MRI Study in Subjects at Ultrahigh Risk of Psychosis Schizophr Bull 2011 in press 10.1093/schbul/sbr035 PMC3446227 21518921

[B13] Drevets WC Bogers W Raichle ME Functional anatomical correlates of antidepressant drug treatment assessed using PET measures of regional glucose metabolism Eur Neuropsychopharmacol 2002 12 6 527 44 10.1016/S0924-977X(02)00102-5 12468016

[B14] Mayberg HS Modulating dysfunctional limbic-cortical circuits in depression: towards development of brain-based algorithms for diagnosis and optimised treatment Br Med Bull 2003 65 193 207 10.1093/bmb/65.1.193 12697626

[B15] Smith DJ Cavanagh JT The use of single photon emission computed tomography in depressive disorders Nucl Med Commun 2005 26 3 197 203 10.1097/00006231-200503000-00004 15722900

[B16] Rigucci S Serafini G Pompili M Kotzalidis GD Tatarelli R Anatomical and functional correlates in major depressive disorder: the contribution of neuroimaging studies World J Biol Psychiatry 2010 11 2 Pt 2 165 80 19670087 10.1080/15622970903131571

[B17] Vlassenko A Sheline YI Fischer K Mintun MA Cerebral perfusion response to successful treatment of depression with different serotoninergic agents J Neuropsychiatry Clin Neurosci 2004 16 3 360 3 10.1176/appi.neuropsych.16.3.360 15377745

[B18] Davies J Lloyd KR Jones IK Barnes A Pilowsky LS Changes in regional cerebral blood flow with venlafaxine in the treatment of major depression Am J Psychiatry 2003 160 2 374 6 10.1176/appi.ajp.160.2.374 12562589

[B19] Goldapple K Segal Z Garson C Lau M Bieling P Kennedy S Mayberg H Modulation of cortical-limbic pathways in major depression: treatment-specific effects of cognitive behavior therapy Arch Gen Psychiatry 2004 61 1 34 41 10.1001/archpsyc.61.1.34 14706942

[B20] Bocchetta A Tamburini G Cavolina P Serra A Loviselli A Piga M Affective psychosis, Hashimoto's thyroiditis, and brain perfusion abnormalities: case report Clin Pract Epidemiol Ment Health 2007 3 31 10.1186/1745-0179-3-31 18096026 PMC2235848

[B21] Boelaert K Newby PR Simmonds MJ Holder RL Carr-Smith JD Heward JM Manji N Allahabadia A Armitage M Chatterjee KV Lazarus JH Pearce SH Vaidya B Gough SC Franklyn JA Prevalence and relative risk of other autoimmune diseases in subjects with autoimmune thyroid disease Am J Med 2010 123 2 183 e1-9 20103030 10.1016/j.amjmed.2009.06.030

[B22] Dickson BC Streutker CJ Chetty R Coeliac disease: an update for pathologists J Clin Pathol 2006 59 10 1008 16 10.1136/jcp.2005.035345 17021129 PMC1861744

[B23] Carta MG Carpiniello B Trudu MN Tarquini A Rudas N Aguglia E, Pascolo E La versione italiana della CIDI Simplified, uno studio di accuratezza e riproducibilità Metropoli e oltre 1994 Trieste: Tencati

[B24] American Psychiatric Association Diagnostic and statistical manual of mental disorders 1994 4 Washington DC: APA

[B25] Robins LN Wing J Wittchen HU The composite international diagnostic interview. An epidemiologic instrument suitable for use in conjunction with different diagnostic systems and in different cultures Arch Gen Psychiatry 1988 45 12 1069 77 2848472 10.1001/archpsyc.1988.01800360017003

[B26] Colamussi P Giganti M Cittanti C Dovigo L Trotta F Tola MR Tamarozzi R Lucignani G Piffanelli A Brain single-photon emission tomography with 99mTc-HMPAO in neuropsychiatric systemic lupus erythematosus: relations with EEG and MRI findings and clinical manifestations Eur J Nucl Med 1995 22 1 17 24 10.1007/BF00997243 7698150

[B27] Carta MG Hardoy MC Boi MF Mariotti S Carpiniello B Usai P Association between panic disorder, major depressive disorder and celiac disease: a possible role of thyroid autoimmunity J Psychosom Res 2002 53 3 789 93 10.1016/S0022-3999(02)00328-8 12217453

[B28] Nagamachi S Jinnouchi S Nishii R Ishida Y Fujita S Futami S Kodama T Tamura S Kawai K Cerebral blood flow abnormalities induced by transient hypothyroidism after thyroidectomy, analysis by tc-99m-HMPAO and SPM96 Ann Nucl Med 2004 18 6 469 77 10.1007/BF02984562 15515745

[B29] Fountoulakis KN Iacovides A Grammaticos P St Kaprinis G Bech P Thyroid function in clinical subtypes of major depression: an exploratory study BMC Psychiatry 2004 4 6 10.1186/1471-244X-4-6 15113438 PMC394331

[B30] Carta MG Hardoy MC Carpiniello B Murru A Marci AR Carbone F Deiana L Cadeddu M Mariotti S A case control study on psychiatric disorders in Hashimoto disease and Euthyroid Goitre: not only depressive but also anxiety disorders are associated with thyroid autoimmunity Clin Pract Epidemol Ment Health 2005 1 23 10.1186/1745-0179-1-23 PMC1308833 16285879

[B31] Fountoulakis KN Kaprinis SG Iacovides A Phokas K Kaprinis G Are dexamethasone suppression test nonsuppression and thyroid dysfunction related to a family history of dementia in patients with major depression? An exploratory study Can J Psychiatry 2005 50 6 342 5 15999950 10.1177/070674370505000609

